# Mammary myofibroblastoma in the right lateral abdominal wall

**DOI:** 10.1186/s12957-016-0796-6

**Published:** 2016-02-24

**Authors:** Jiyong Pan, Shuang Wang, Yingyi Zhang, Zhe Fan

**Affiliations:** Department of General Surgery, the Third People’s Hospital of Dalian (Dalian Third People’s Hospital Affiliated to Dalian Medical University), Dalian, 116033 China; VIP Department, Affiliated Zhongshan Hospital of Dalian University, Dalian, 116001 China

**Keywords:** Extramammary, Myofibroblastoma, Abdominal wall

## Abstract

**Background:**

A mammary-type myofibroblastoma is a rare soft tumor; extramammary myofibroblastomas are especially rare.

**Case presentation:**

A 51-year-old woman presented to our department for evaluation of a mass on the right lower abdominal wall. The mass was then excised completely. Gross examination showed a huge, well-circumscribed soft tissue mass. The pathologic diagnosis was an extramammary myofibroblastoma. There was no recurrence after excision at the 6-month follow-up visit.

**Conclusions:**

A mammary-type myofibroblastoma is a benign soft tissue neoplasm. No malignant behavior and/or recurrence of mammary-type myofibroblastomas after surgical resection have been described as a function of size and location. The present case aimed to provide a possible differential diagnosis for such abdominal masses.

## Background

Mammary myofibroblastomas are benign tumors, which were first described in the breast by Wargotz et al. in 1987 [1]. Extramammary myofibroblastomas have been reported more frequently. Previous reports [2, 3] have suggested that the inguinal/groin region is the usual site for extramammary myofibroblastomas. The tumor usually consists of spindle cells [4] and lacks genetic material in the 13q14 region [5]. This is the second case report of a myofibroblastoma on the abdominal wall. This study was approved by the hospital’s Ethics Committee

## Case presentation

**Fig. 1:**
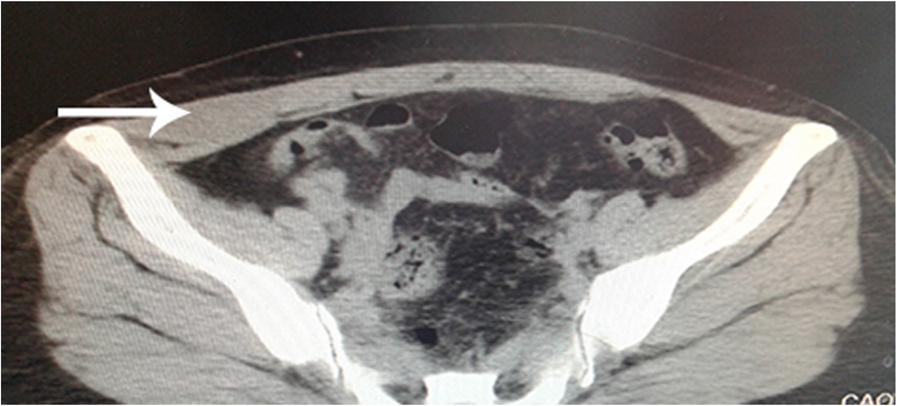
Compared with the left side, thickened muscle was noted on the right lateral abdominal wall. There was no clear limit of the mass

**Fig. 2:**
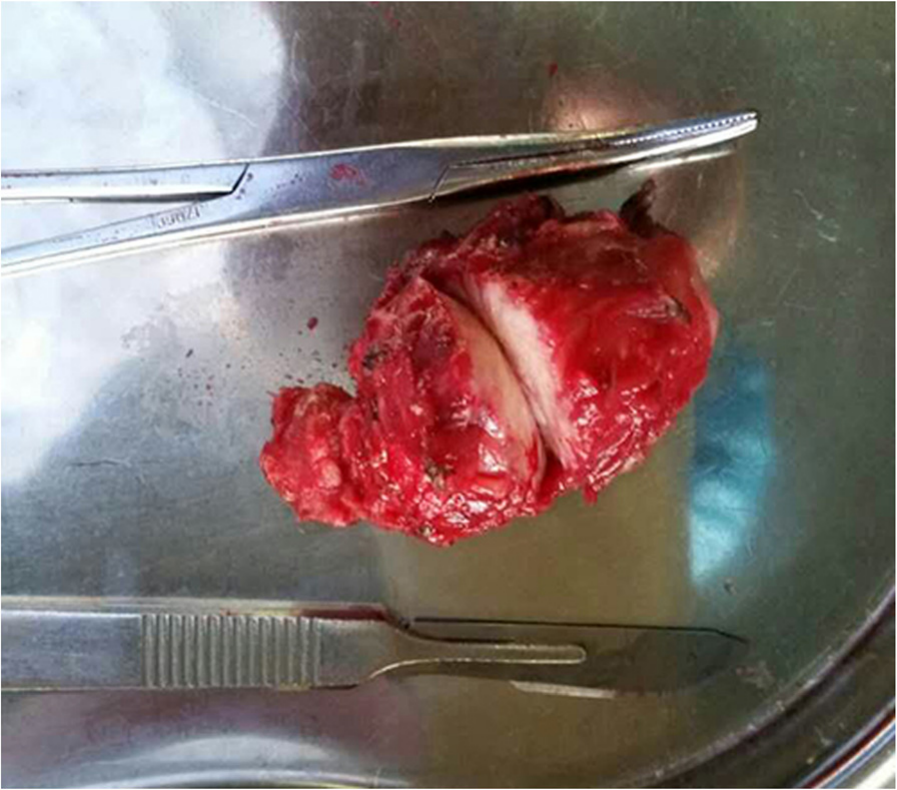
A white-to-gray, well-circumscribed, firm mass, approximately 2 × 3 × 4 cm in size

A 51-year-old female presented to our department (the Third People’s Hospital of Dalian, Dalian, China) complaining of a mass on the right lower abdominal wall that had been there for 20 years. The patient had normal vital signs. The mass on the right lateral abdominal wall was located through physical examination of the abdomen. The mass was approximately 2 × 4 cm in diameter, well-circumscribed, firm, and painless. Laboratory testing (blood and biochemistry routine examination) was normal. Computed tomography (CT; Fig. 1) revealed thickened muscle of the right lateral abdominal wall. Thus, surgical excision was recommended. Intra-operatively, a mass was noted in the right aspect of the internal oblique muscle of the abdomen with no invasion of the underlying tissues. A complete resection of the mass was performed. The tumor was well-circumscribed, firm, and measured approximately 2 × 3 × 4 cm (Fig. 2). The tumor had a dusty pink color and resilient characteristics. A frozen section showed a spindle cell tumor, and pathologic evaluation of the tumor revealed an extramammary myofibroblastoma. Immunohistochemistry revealed that the mass was smooth muscle actin (SMA)-positive, S-100-negative, CD34-negative, and the Ki67+ <1 %. The patient was followed for 6 months, and no recurrence was noted.

### Discussion

A mammary myofibroblastoma is a benign tumor that was first described in the breast [1]. Mammary myofibroblastomas are well-circumscribed with abundant fascicular clusters of spindle cells as the main component [6]. Extramammary myofibroblastomas are rare. McMenamin [5] has reported a case of abdominal wall myofibroblastoma. Extramammary myofibroblastomas often originate from the embryonic milk line, similar to accessory breasts [5]. Extramammary myofibroblastomas are most often found in the iliac region [3] and rarely in the retroperitoneum, axilla, posterior vaginal wall, mid-back, left buttocks, paratesticular region [5], seminal vesicle [7], and perianal region [8].

Extramammary myofibroblastomas have a predilection for older men, post-menopausal women, children, and adolescents [1]. Abdul-Ghafar [2] reported that the average age of patients with extramammary myofibroblastomas was 52.5 years. The typical symptoms in patients with extramammary myofibroblastomas include slowly growing painless tumors; few patients report pain. The diameter of extramammary myofibroblastoma ranges from 1 to 4 cm [1, 5].

The tumor is usually well-demarcated, and the color is white-to-gray. A rubbery consistency with a thin fibrous capsule and hemorrhagic areas have also been described [9].

The histopathologic changes associated with extramammary myofibroblastomas often include abundant fascicular clusters of spindle- and oval-shaped cells, which are arranged in interlaced or swirled patterns [4]. The above characteristic was also detected in our patient.

Ultrasound often demonstrates a solid structure with mixed echogenicity or lobulated margins in patients with extramammary myofibroblastomas [10]. CT can also be performed to exclude the possibility of a different origin or involvement in proximal structures [3]. In the present case, CT revealed hyperplastic muscle of the right lateral abdominal wall, which was not symmetric with the contralateral side.

Xu et al. [4] indicate that CT and MRI are useful imaging methods in the diagnosis of myofibroblastoma. Shinojima et al. [11] described myofibroblastoma as a low- or mixed low- and high-density mass on CT, which was similar with CT of our present patient. Because of the patient’s reason, MRI was not performed. Shinojima et al. [11] refer that the myofibroblastoma was isointense on T1- and hypointense on T2-weighted MI. The absence of MRI resulted in an imperfect evaluation. Therefore, when doctors encounter a tumor like this, examination about that should be carried out as far as possible, including ultrasound, CT, and MRI, even fine needle aspiration.

The tumor is usually shown to be positive for smooth muscle actin, muscle-specific actin, and vimentin and negative for c-kit, carcinoembryonic antigen, keratins, S-100, human melanoma black-45 (HMB-45), and Epstein-Barr virus latent membrane protein 1. Reactivity to factor VIII-related antigen (a marker of endothelial cells), EMA, MAK-6 (cytokeratin; a marker of epithelial cells and meningeal cells), and glial fibrillary acidic protein is negative [3, 12, 13]. Extramammary myofibroblastomas are weakly positive for desmin and CD34 [4, 11]. In the present case, immunohistochemical staining was SMA-positive, S-100-negative, CD34-negative, and Ki67+ <1 %.

The differential diagnosis of this tumor includes the following: Kaposi’s sarcoma, leiomyosarcoma, intranodal schwannoma, benign metastasizing leiomyoma (BML), follicular dendritic cell sarcoma (FDCS), inflammatory myofibroblastic tumor (IMT), metastatic malignant melanoma, and metastatic carcinoma [9].

Kaposi’s sarcoma is primarily a borderline vascular tumor implicating the skin and mucosa, but in more advanced stages, the lymph nodes and almost any internal organ could also be involved; human herpesvirus 8 (HHV8) is considered the causative agent for Kaposi’s sarcoma [14], while myofibroblastomas usually spring from the embryonic milk line and locate at mamma.

Leiomyosarcoma is a malignant mesenchymal tumor with smooth muscle origin that represents 5–7 % of all soft tissue sarcomas [15], which is different from the origin of myofibroblastomas. Intranodal schwannoma means schwannoma arising in lymph nodes, reclassified as palisaded myofibroblastomas [16, 17].

Intranodal schwannoma were often positive for vimentin and S100, which are similar with myofibroblastomas, and negative for SMA and CD34, which are not similar with myofibroblastomas. The findings of intranodal schwannoma were consistent with a diagnosis of primary schwannoma of the lymph node [17].

BML is a rare neoplastic process in which of the uterine leiomyomas metastasize to distant sites, the most common of which are the lungs [18], and the patients often are treated with hysterectomy [19]. The difference between BML and myofibroblastomas is their common location.

Dendritic cell sarcoma (DCS) been previously described by many terms such as lymphoma, sarcoma, or histiocytic neoplasm, reflecting the controversy about the tumors [20]. FDCS is specifically immunopositive to CD21, CD35 and/or CD23, vimentin, fascin, HLA-DR, EMA, D2-40, clusterin, and CXCL13 [21].

IMT is a rare mesenchymal tumor and has been considered an inflammatory condition; the presentations of neoplastic proliferation include invasive potential, recurrences, and metastases [22]. It is mostly located in the lungs, then in the abdomen (especially in the mesentery), and other parts such as the liver, bladder, and stomach. The clinical presentation depends on its location [23].

Metastatic malignant melanoma and metastatic carcinoma could metastasize to any organ or multiple organs. As typical symptoms are not specific and show organ-specific outcomes depending on metastasized lesions, it is difficult to be suspected at the onset [24]. Myofibroblastoma have a well-circumscribed degree different from metastatic malignant melanoma and metastatic carcinoma.

## Conclusions

Extramammary-type myofibroblastomas are benign soft masses without malignant potential. Surgical resection is the preferred method for cure, and the recurrence rate is low. As an extremely rare tumor, physicians should pay more attention to differential diagnosis.

### Consent

Written informed consent was obtained from the patient for publication of this case report and the accompanying images.

## Abbreviations

SMA: smooth muscle actin
HMB-45: human melanoma black-45.
